# Centre Selection for Clinical Trials and the Generalisability of Results: A Mixed Methods Study

**DOI:** 10.1371/journal.pone.0056560

**Published:** 2013-02-22

**Authors:** Adrian Gheorghe, Tracy E. Roberts, Jonathan C. Ives, Benjamin R. Fletcher, Melanie Calvert

**Affiliations:** 1 Primary Care Clinical Sciences, University of Birmingham, Birmingham, United Kingdom; 2 Health Economics Unit, University of Birmingham, Birmingham, United Kingdom; 3 MRC Midland Hub Trials Methodology Research, University of Birmingham, Birmingham, United Kingdom; University Paris Descartes, France

## Abstract

**Background:**

The rationale for centre selection in randomised controlled trials (RCTs) is often unclear but may have important implications for the generalisability of trial results. The aims of this study were to evaluate the factors which currently influence centre selection in RCTs and consider how generalisability considerations inform current and optimal practice.

**Methods and Findings:**

Mixed methods approach consisting of a systematic review and meta-summary of centre selection criteria reported in RCT protocols funded by the UK National Institute of Health Research (NIHR) initiated between January 2005-January 2012; and an online survey on the topic of current and optimal centre selection, distributed to professionals in the 48 UK Clinical Trials Units and 10 NIHR Research Design Services. The survey design was informed by the systematic review and by two focus groups conducted with trialists at the Birmingham Centre for Clinical Trials. 129 trial protocols were included in the systematic review, with a total target sample size in excess of 317,000 participants. The meta-summary identified 53 unique centre selection criteria. 78 protocols (60%) provided at least one criterion for centre selection, but only 31 (24%) protocols explicitly acknowledged generalisability. This is consistent with the survey findings (n = 70), where less than a third of participants reported generalisability as a key driver of centre selection in current practice. This contrasts with trialists’ views on optimal practice, where generalisability in terms of clinical practice, population characteristics and economic results were prime considerations for 60% (n = 42), 57% (n = 40) and 46% (n = 32) of respondents, respectively.

**Conclusions:**

Centres are rarely enrolled in RCTs with an explicit view to external validity, although trialists acknowledge that incorporating generalisability in centre selection should ideally be more prominent. There is a need to operationalize ‘generalisability’ and incorporate it at the design stage of RCTs so that results are readily transferable to ‘real world’ practice.

## Introduction

Randomised controlled trials (RCTs) have long been the gold standard research design because of their potential to offer unbiased estimates of interventions’ effectiveness. The external validity (generalisability) of trial results in routine clinical practice and their relevance for health policy makers may, however, be questioned [1]–[3]. For example, evidence suggests that trial participants are often unrepresentative of the target population [4]–[9], which can introduce bias in the measures of effect [10]. Various trial designs [11]–[13], recommendations [14] and tools [15] have been suggested to enhance or assess the applicability of RCTs.

The choice of participating centres can also influence the generalisability of trial results [2], especially in non-pharmacologic trials, as outcomes may be affected by factors like hospital volume [16] and practitioners’ expertise [13]. For example, the systematic review of Halm et al [16] found that patients treated in higher volume hospitals have better clinical outcomes across a wide range of therapeutic areas. In surgical RCTs, restricting participation to centres where surgeons have a proven record of success may lead to results which depart greatly from real-life estimates [2]. Practice guidelines can also differ from one hospital to another. For instance, negative pressure wound therapy (NPWT) is a technology currently used in the UK for the open abdomen at the discretion of NHS trusts in the absence of a nationwide recommendation towards its implementation [17]. Limited evidence suggests that RCTs are predominantly carried out in university and teaching centres, while non-teaching centres are somewhat better represented in non-randomised studies [18]. The influence of centre-specific characteristics on treatment outcomes has been equally recognized in observational research [19]. Ensuring the generalisability of RCT results may be particularly challenging for economic outcomes informing health policy changes. Whilst the relative clinical effect of an intervention has been historically assumed constant across settings, albeit not without challenges [2], [20], [21], this assumption may not hold for economic outcomes [22], [23]. Modelling methods are one way to retrospectively address this limitation, but rely on inferences made on a sample of centres, which may not be representative within the country to which the policy decision will apply [24]–[28].

Further, it is often difficult to ascertain the generalisability of RCT results since reporting in trial publications is poor [29]–[31]. As a recent development, the extension of the CONSORT statement to randomised trials of nonpharmacologic treatments requires discussing generalisability in relation to the care providers and centres involved in the trial [32].

Given that the sample of participating centres may impact on the generalisability of trial results, especially with respect to decision making based on cost-effectiveness evidence, the question arises as to whether the current practice of clinical trials design and conduct allows for such a bias to occur. Our research had two objectives: first, to establish which factors currently drive centre selection in trials; and second, to reveal what is perceived as good practice in terms of enrolling centres.

## Methods

### Ethics Statement

The Science, Technology, Engineering and Mathematics Ethical Review Committee at the University of Birmingham have favourably reviewed this study (Focus group Ref. no. ERN_11-0792 and survey Ref. no. ERN_11-1347). No ethical approval was necessary for the systematic review. Focus group participants and survey respondents were asked for informed consent prior to their participation in the study. Focus group participants provided written informed consent on forms that have been approved by the Ethical Review Committee.

A mixed methods approach was employed: we conducted a systematic review of protocols of RCTs funded by the National Institute for Health Research - Health Technology Assessment (NIHR-HTA) programme; and we surveyed professionals in the 48 UK Clinical Research Collaborative Clinical Trials Units (UKCRC CTUs) and 10 NIHR Research Design Services (RDS). The survey was informed by the systematic review and two focus groups with experienced trialists.

We targeted RCTs conducted with a clear view to influence policy and thus included studies with a built-in economic evaluation funded by the UK NIHR-HTA stream. The systematic review was complemented with a survey of trialists for the following reasons: 1) there is a large amount of heterogeneity in the structure of HTA trial protocols and reporting criteria for selecting sites/clinicians is not a pre-requisite, leaving it at the choice of the researchers; 2) there is evidence in the literature on poor adherence to trial protocols [33], [34]; and 3) there is no guarantee that the trialists involved in writing the (sections relevant for centre selection of the) protocol are the ones who actually perform the selection in practice, potentially bringing new considerations in the process. Considering all the above, we aimed to obtain a first-hand account from trialists and compare it with the findings of the systematic review. We contrasted current and optimal practice in order to explore trialists’ views on the extent to which generalisability in centre selection should be explicitly considered in trial design.

### Systematic Review

The systematic review aimed to identify the reported rationale for including centres in RCTs. We reviewed full-length protocols of all the RCTs included in the NIHR HTA Primary Research portfolio initiated between January 2005 and January 2012. No search terms were used as all the projects available in the portfolio were assessed.

The inclusion criteria were: multi-centre RCTs with an explicit economic evaluation component and at least one centre recruited from the UK. The following studies were excluded: single centre RCTs; pilot RCTs, feasibility studies and follow-up studies; any non-randomised study i.e. observational studies, diagnostic tests studies and analytic decision models based on one or more RCTs; studies without an explicit economic evaluation; and projects for which the full-length trial protocol was not available.

The following data were extracted from each included study: name of chief investigator; project start year; therapeutic area; study design; evaluated interventions; type of intervention (pharmacologic vs. non-pharmacologic); type of comparator (placebo vs. usual care or another intervention); and information on centre selection (free text). The free text was analyzed using the meta-summary method [35], such that the information was abstracted, reformulated and categorized. A frequency effect size was calculated for each emerging category as the ratio between the number of studies containing a finding and the total number of included studies.

Study selection and data extraction were performed by one researcher (AG) and a random sample of 20% of studies was checked by another (BF). The meta-summary was performed by one researcher (AG) and reviewed entirely by another (BF). The protocol of the systematic review is available in Appendix S1.

### Focus Groups

Invitations to participate in focus groups were circulated to all staff affiliated with the Birmingham Centre for Clinical Trials, comprising three distinct trials units: Cancer Research UK Trials Unit, Birmingham Clinical Trials Unit and Primary Care Clinical Research Trials Unit. We conducted two focus groups (n = 6 and 4 participants, respectively) exploring trialists’ thoughts and experiences of centre selection with the following aims: first, to identify potential reasons for centre selection not already identified in the systematic review; and second, to derive a meaningful conceptual framework informing the design of the online survey. The focus group participants included the following professionals: clinical investigator (n = 1), trial managers (n = 5), health economist (n = 1), statistician (n = 1) and trial methodologists (n = 2).

Focus group methodology was useful here because it allowed the capture of data that resulted from discussion and negotiation [36], and thus helped distinguish between factors that affected participants as a group and those that were specific to individuals. Discussions were structured using a topic guide that ensured key issues were explored [37], but participants were able to direct the content of the discussion, allowing unanticipated themes to arise. Discussions were transcribed verbatim and analyzed using simple conventional content analysis [38], in which the data were coded and arranged into meaningful organizational units, from which themes were derived that described the participants’ views. The analysis was performed by one researcher (AG) and reviewed entirely by another (JI).

### Survey

The considerations emerging from the systematic review and the focus groups informed an online survey with two sections: the first section asked the respondents about the *current practice* of centre selection for RCTs in terms of influential considerations and key professionals involved in the process; and the second section used the same questions to elicit respondents’ views about what should constitute *optimal practice* (Appendix S2). Respondents were asked to assume that the minimum centre requirements for participation in the trial were met i.e. access to the study population and required time, staff and facilities for running the RCT.

The participants were asked to choose from a comprehensive list a minimum of three and a maximum of five items they considered to be most important for centre selection. No explicit ranking was required. The respondents’ views on the role of health economics considerations in centre selection were also explored. Prior to distribution the survey was piloted with the focus group participants, who commented on its structure and content.

A secure link to the survey was distributed by email to the directors and deputy directors of all 48 UKCRC CTUs and ten NIHR RDS, who were invited to complete the questionnaire and forward it to relevant staff within their units. ‘Relevant staff’ explicitly referred to: clinical investigators, trial coordinators/trial managers, statisticians, health economists and any other academic position (e.g. research associate, research fellow). One reminder email was circulated two weeks after the initial distribution. Only the complete responses were included in the analysis, which was performed using STATA 10 software (StataCorp, College Station TX, US).

The survey was anonymous: the only personal information items referred to the participants’ professional role and their experience (years) in the design and/or conduct of RCTs.

## Results

### Systematic Review

**Figure 1:**
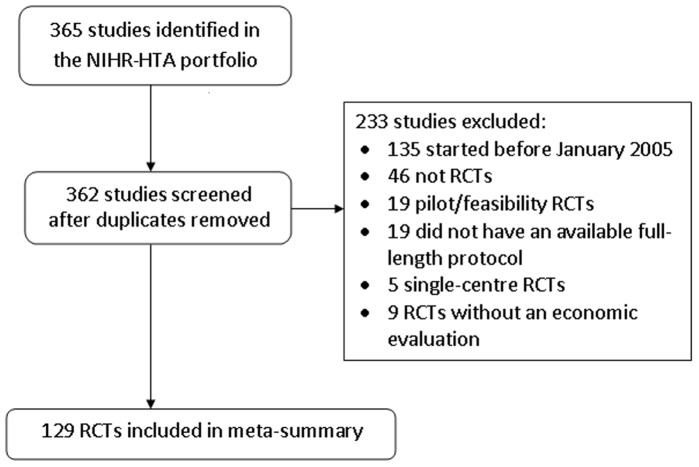
Systematic review: Study inclusion flowchart.

We reviewed 365 projects in the UK NIHR HTA Primary Research portfolio of which 129 RCTs met the inclusion criteria, with a target sample size total of more than 317,000 participants (Figure 1). The majority of included RCTs had a parallel design (n = 112; 87%) and investigated non-pharmacologic interventions (n = 96; 74%). Table 1 presents a descriptive summary of the included studies.

**Table 1:** Systematic review: Characteristics of included RCTs.

Characteristic	Number of studies (%, n = 129)
**International recruitment**	
Yes	9 (7%)
No	120 (93%)
**Design**	
Parallel	112 (87%)
Cluster	14 (11%)
Factorial	3 (2%)
**Intervention**	
Pharmacologic intervention	33 (26%)
Non-pharmacologic intervention	96 (74%)
**Comparator**	
Placebo	9 (7%)
Standard care or other intervention(s)	120 (93%)
**Therapeutic area**	
Mental health	25 (19%)
Oncology	9 (7%)
Musculoskeletal disorders	9 (7%)
Respiratory disorders	8 (6%)
Obstetrics and gynaecology	8 (6%)
Behavioural medicine	8 (6%)
Neurology	7 (5%)
Infectious diseases	6 (5%)
Digestive tract disorders	6 (5%)
Cardiology	6 (5%)

Other therapeutic areas with less than 5% of studies were (number of studies): obesity (5), diabetes (5), urology (5), haematology (5), circulatory disorders (5), dermatology (3), dentistry (3), emergency medicine (2), ageing (2) and five other miscellaneous areas.

There were 78 studies (60%) reporting one or more criteria related to centre selection. The meta-summary identified 53 unique considerations (Table S1) that were grouped into three themes (Table 2).

**Table 2:** Systematic review: Centre selection considerations, results of the meta-summary - subgroup analysis by type of intervention and RCT design.

	Frequency (effect size)				
Themes	Total	Non-pharmacologic	Pharmacologic	Cluster	Non-cluster
	(n = 129)	RCTs (n = 96)	RCTs (n = 33)	RCTs (n = 14)	RCTs (n = 115)
**PROVIDED CONSIDERATIONS FOR CENTRE SELECTION**	**78 (60%)**	**56 (58%)**	**22 (67%)**	**13 (93%)**	**65 (57%)**
**Diversity and representativeness in terms of…**	**31 (24%)**	**26 (27%)**	**5 (15%)**	**6 (43%)**	**25 (22%)**
Population characteristics	14 (11%)	13 (14%)	1 (3%)	2 (14%)	12 (10%)
Health service delivery	15 (12%)	13 (14%)	2 (6%)	6 (43%)	9 (8%)
Centre setting	15 (12%)	12 (13%)	3 (9%)	2 (14%)	13 (11%)
**Centre characteristics**	**57 (44%)**	**39 (41%)**	**18 (55%)**	**7 (50%)**	**50 (43%)**
Centre setting	4 (3%)	3 (3%)	1 (3%)	2 (14%)	2 (2%)
Health service delivery (‘research-ready’)	16 (12%)	11 (11%)	5 (15%)	0 (0%)	16 (14%)
Trial intervention	31 (24%)	24 (25%)	7 (21%)	3 (21%)	28 (22%)
Research	19 (15%)	11 (11%)	8 (24%)	2 (14%)	17 (15%)
Centre size (catchment area/patient throughput)	22 (17%)	16 (17%)	6 (18%)	4 (29%)	18 (16%)
Trial participation	37 (29%)	23 (24%)	14 (42%)	8 (57%)	29 (25%)
Recruitment	17 (13%)	10 (10%)	7 (21%)	3 (21%)	14 (12%)
**Trial constraints (time, budget)**	**5 (4%)**	**5 (5%)**	**0 (0%)**	**4 (29%)**	**1 (1%)**
Ensuring trial processes and requirements	24 (19%)	13 (14%)	11 (33%)	3 (21%)	21 (18%)
Support for running the trial	7 (5%)	6 (6%)	1 (3%)	3 (21%)	4 (3%)
Willingness	9 (7%)	7 (7%)	2 (6%)	1 (7%)	8 (7%)

Model interpretation: Out of 26 non-pharmacologic RCTs (27% of non-pharmacologic trials) which mentioned at least one consideration for centre selection pertaining to diversity and representativeness, 13 RCTs (14%) were concerned with diversity in terms of population characteristics, 13 RCTs (14%) mentioned diversity in terms of health service delivery and 12 RCTs (13%) referred to diversity in terms of centre setting.

### Theme 1: ‘Diversity and Representativeness’

In 31 studies (24%) the rationale for centre selection included the need for a diverse or representative sample. Representativeness could be categorised as ‘population characteristics’ such as ethnicity or socioeconomic status (n = 14; 11%), ‘health service delivery’ in terms of case-mix, or throughput (n = 15; 12%) and ‘centre setting’ in terms of size or geographical location (n = 15; 12%).

### Theme 2: ‘Centre Characteristics’

In 57 studies (44%) the rationale for centre selection included pragmatic characteristics categorised as ‘centre setting’, ‘health service delivery (research ready)’, ‘intervention’, ‘research’ and ‘centre size’. In some trials centres were selected based on investigators’ preference for a particular geographical region (e.g. proximity to the academic institution leading the study). Other pragmatic reasons included whether the centre was ‘research ready’ in terms of holding official certifications, links with relevant facilities or running computerized prescribing systems. 31 (24%) of protocols described considerations related to the intervention(s) under investigation in the RCT e.g. centres were required to have had training, experience and/or a proven record of implementing the intervention. In other cases, intervention-naive centres were explicitly preferred. Interest in ‘research’ manifested in targeting sites with research experience or that were part of an established research network. 22 (17%) studies targeted centres of a particular size (Table 2).

Complete results of the meta-summary are available in Table S1.

### Theme 3: ‘Trial Participation’

In total 37 studies (29%) reported further pragmatic reasons for centre selection which focused on the ability of a centre to deliver the trial successfully within the time frame and budgetary constraints (Table 2).

### Focus Groups

The analysis of the focus groups identified three categories of considerations relevant for centre selection: minimum centre requirements (access to the relevant study population, willingness to participate in the trial and availability of resources such as time, staff and facilities), desirable centre characteristics and other considerations e.g. the state of the local research environment and patient convenience.

### Survey

We received 70 complete responses to the survey. Trial managers were the best represented professionals (n = 21; 30%) and most respondents (n = 49; 70%) had been involved in the design and/or conduct of RCTs for more than five years (Table 3).

**Table 3:** Survey: Profile of survey participants.

Characteristic	Respondents (%, n = 70)
**Professional role**	
Clinical investigator	9 (13%)
Statistician	13 (19%)
Trial coordinator	21 (30%)
Health economist	5 (7%)
Clinical trials methodologist	7 (10%)
Epidemiologist	1 (1%)
Other academic position	7 (10%)
Other professionals	7 (10%)
**Experience in design/conduct of RCTs**	
Less than 2 years	3 (4%)
Between 2 and 5 years	18 (26%)
Between 5 and 10 years	19 (27%)
More than 10 years	30 (43%)

For both current and optimal practice, respondents indicated the most desirable centre characteristics, the factors with the largest influence on centre selection and the key individuals driving the selection process. In current practice, the most desirable centre characteristics were: the ability to recruit patients, centre staff displaying interest in the RCT and good communications with the trial office (Table 4). Most respondents reported that including a centre in a RCT is influenced by the centre staff’s motivation to participate in the RCT (n = 52; 74%) and the local research environment i.e. trial fatigue and competing trials (n = 48; 69%). Ensuring generalisability in terms of population characteristics and clinical practice were mentioned by 33% (n = 23) and 29% (n = 20) of respondents, respectively, while 7% (n = 5) of them referred to the generalisability of economic evaluation results. The trial coordinator and the chief investigator appear to be the key drivers in the process of centre selection. 25% of respondents reported that health economics considerations have a limited influence in centre selection, while 75% reported no such influence.

**Table 4:** Survey: Current and optimal centre selection for RCTs (n = 70).

	Current		Optimal	
Survey questions	practice		practice	
	N	%	N	%
**1. Desirable centre characteristics**				
Ability to recruit patients	61	87%	52	74%
Understanding RCTs	10	14%	16	23%
Good communication with trial office	37	53%	26	37%
Convenient geographical location	17	24%	3	4%
Having support from local commissioners	16	23%	10	14%
Part of a relevant research network	11	16%	9	13%
Ability to obtain necessary approvals timely	33	47%	25	36%
Showing interest in the RCT	44	63%	28	40%
Computer systems are compatible with the trial centre	4	6%	1	1%
Retains/contributes to generalisability (population characteristics)	23	33%	40	57%
Retains/contributes to generalisability (clinical practice)	20	29%	42	60%
Retains/contributes to generalisability (economic evaluation)	5	7%	32	46%
Centre staff have experience with conducting RCTs	28	40%	23	33%
**2. Considerations influencing the process of centre selection**				
Centre staff are motivated to participate	52	74%	41	59%
Centre staff know the Chief Investigator	29	41%	4	6%
Geographical setting (rural vs. urban)	8	11%	18	26%
Requirements of funding/regulatory bodies	13	19%	14	20%
State of local research environment	48	69%	24	34%
Recruiting time frame of the RCT	27	39%	31	44%
Budget of the RCT	21	30%	14	20%
Efficiency of local R&D department	26	37%	17	24%
Disease rarity	9	13%	17	24%
Trial-design characteristics	40	57%	52	74%
Patient convenience	6	9%	22	31%
**3. Professionals driving the process of centre selection**				
Chief Investigator	38	54%	19	27%
Trial coordinator/Trial manager	45	64%	33	47%
Research networks	16	23%	24	34%
Trial statistician	0	0%	1	1%
Trial health economist	1	1%	6	9%
Trial Management Group members as a team	25	36%	41	59%
Data Monitoring Committee members	0	0%	2	3%

In optimal practice, the majority of survey participants indicated the ability to recruit (n = 52; 74%) as desirable, followed by ensuring generalisability in terms of clinical practice (n = 42; 60%), population characteristics (n = 40; 57%) and economic evaluation results (n = 32; 46%), respectively. Most respondents indicated that trial-design characteristics e.g. sample size and number of centres required, and centre staff motivation for the RCT should influence centre selection. Trial management group members as a team should ideally drive centre enrolment.

## Discussion

### Summary of Main Findings

The results of the systematic review and survey consistently suggest that trialists’ decisions regarding centre selection in RCTs are driven by pragmatic considerations, such as: the ability to recruit patients, the centre staff’s motivation to participate and ensuring good communication. The findings suggest that generalisability of RCT results does not appear to drive centre selection: 40% (n = 51) of the reviewed RCTs did not report any rationale for selecting the participating centres and only 31 studies (n = 24%) explicitly acknowledged diversity or representativeness when including centres. Similarly, enrolling centres that ensure the generalisability of results in terms of population characteristics and clinical practice was relevant in current practice for 33% (n = 23) and 29% (n = 20) of survey respondents, respectively. In optimal practice, however, more than 50% of survey respondents were interested in ensuring generalisability.

Both the systematic review and the survey showed that trialists are primarily interested in centres that they trust to recruit well and meet intervention-related requirements based on prior experience and proven performance. Trialists are keen to recruit highly motivated centres, as illustrated by the focus group and survey results.

**Figure 2:**
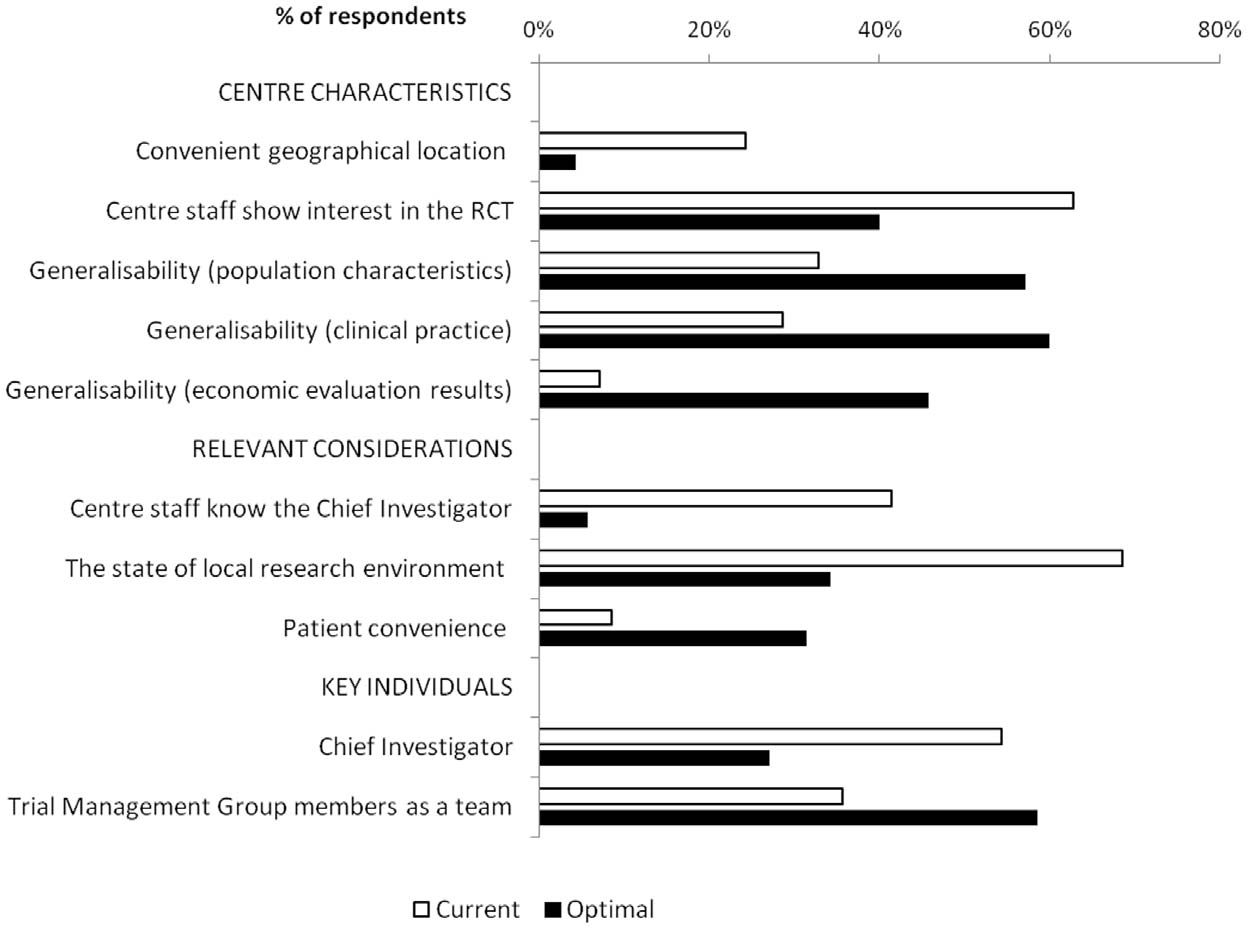
Survey: Discrepancies between the current and optimal practice of centre selection for RCTs.

There are discrepancies between current and optimal centre selection (Figure 2). In current practice centres are often enrolled based on pragmatic reasons such as: convenient location in relation to the trial office, or targeting of sites where centre staff are known to the Chief Investigator. Trialists acknowledge that in optimal practice centre selection should consider more factors such as: ensuring the generalisability of trial results; accounting for patient convenience or making centre enrolment decisions on a collective basis within the trial team. For example, the centre staff knowing the Chief Investigator appears to be important in current practice (n = 29; 41%), but this is not the case in optimal practice (n = 4; 6%). On the other hand, concern for patient convenience when selecting centres is currently rare (n = 6; 9%), but should ideally be more prominent (n = 22; 31%).

There is little evidence that centres normally included in RCTs are purposively chosen to be representative of their jurisdictions or that the selection process is consistent across RCTs. Only two studies included in our systematic review selected sites randomly from a pre-specified pool.

### Strengths and Limitations

A key strength of our study is that it investigated the practice of centre selection from different perspectives and took a mixed methods approach to validate the findings, which appear consistent across methods. Moreover, this research explored the rationale underpinning centre selection, thus contributing to the understanding of conflicting tensions in trial conduct and design, as perceived by trialists. Furthermore, both the focus groups and the survey presented the views of a wide range of professionals involved in the design and conduct of clinical trials, thus contributing to the validity of our findings.

The choice for a mixed methods approach was founded upon the aim to build a comprehensive picture of the current practice of centre selection in trials. We chose to look at trials funded through the NIHR HTA Primary Research programme as this background gives them a clear view to influencing nationwide health policy. For this reason we only included trials with an explicit economic evaluation component, as an economic assessment is currently a pre-requisite for national policy changes in the UK. Furthermore, the potential for centre-induced bias is the greatest for economic outcomes.

One potential limitation of this research is that the systematic review considered RCTs in the UK NIHR HTA portfolio, of which only 7% recruited internationally. Nevertheless, the size of our sample and the coverage of diverse therapeutic areas contribute to the relevance of our findings. It is possible that centre selection is more thoroughly addressed in trials conducted in other countries; however there is little evidence to support this. Furthermore, our meta-summary concentrated on the explicit reporting of including centres and did not aim to generate a standalone theory on centre selection, for which a more in-depth qualitative technique would have been more appropriate. While the RCTs included in the review were publicly-funded, which can be regarded as a limitation, it can equally be argued that generalisability was of interest to the funder given the view to influence nationwide policy. This assumption may not hold for pharmaceutically-sponsored RCTs and generalisability may actually be worse accounted for than our findings show when the entire spectrum of public-private RCTs are considered.

Our systematic review included trials with an explicit economic evaluation component. While this may be viewed as a limitation, two further points must also be considered. Firstly, the UK decision making body i.e. the National Institute for Health and Clinical Excellence (NICE) requires evidence of cost-effectiveness before advising on the nationwide adoption of a medical technology. The economic evaluation component is therefore mandatory for such policy changes, which makes it extremely relevant in the context of generalisability and we attempted to incorporate it accordingly. Furthermore, and lending strength to the previous consideration, only 9 trials out of the 365 trials considered were excluded from our systematic review because they did not have an explicit economic evaluation component (Figure 1). This suggests that their exclusion is unlikely to have biased our sample and confirms that most UK trials do indeed evaluate economic outcomes.

It can be argued that the potential bias associated with centre selection is of far more relevance for some trials (e.g. primary care and surgery trials) than for others (e.g. drug trials), therefore our meta-summary may have overestimated the extent of centre selection misreporting by pooling together various types of RCTs. However, our sample was dominated by non-pharmacologic trials and an exploratory subgroup analysis (Table 2) revealed that pharmacologic trials actually did better than non-pharmacologic trials in reporting centre selection considerations (67% vs. 58%), but, as expected, were less concerned with generalisability (15% vs. 27%). We acknowledge that our study sample included a high proportion of non-pharmacologic trials, which may limit the applicability of our findings.

We also performed an exploratory sub-group analysis investigating the differential reporting of centre selection considerations in cluster RCTs and non-cluster RCTs, respectively (Table 2). The effect sizes suggest that cluster RCTs perform better than non-cluster RCTs in reporting centre selection considerations (93% vs. 57%), especially in relation to representativeness (43% vs. 22%) and trial participation (57% vs. 25%). While such a finding does not come as a surprise given the obvious interest of accounting for setting-dependent effects in cluster trials, the small number of such RCTs in our sample i.e. 14 out of 129, preclude any strong inferences to be made.

Finally, while a priming effect is possible when comparing current and ideal practice in the survey, the results are not consistent with such an effect: the centre’s ability to recruit patients and staff’s motivation to participate in the RCT were most prominent both in current and optimal practice, which testifies their importance for trialists. On the other hand, the largest relative increase in importance from current to optimal practice was for the three generalisability items. A response rate for the survey could not be calculated as completion relied on trials units’ directors distributing the survey link to their staff, given that individual contact details were not available.

### Relation to Other Studies

Results presented here are in line with previous research indicating that reporting generalisability in trials is sub-optimal. The systematic review of Braslow et al [29] included 414 randomised and non-randomised studies published in the area of mental health between 1981 and 1996. Using self-constructed external validity indices, the authors found that 75% of the studies did not address sample representativeness and only 3% employed a random or systematic sampling method. Jacquier et al [39] reviewed 158 surgical RCTs and found that only 64 of them (41%) reported selection criteria for the surgeons performing the interventions. Eldridge et al [31] found in their review of 34 cluster RCTs that 47% of studies did not discuss cluster generalisability.

While previous studies have used original or published checklists to assess generalisability, our study investigated what actually drives the inclusion of centres in RCTs rather than comparing research practice against an external framework. The findings offer a snapshot of what trialists themselves perceive to be important, both in current and optimal practice. As it has been previously suggested [31], the seminal problem with respect to generalisability is that, despite the available assessment frameworks, there is very little guidance as to how the relevant considerations can be incorporated in the trial design and how the degree of generalisability of a trial’s results can be quantified.

### Significance and Relevance

Our findings suggest a large discrepancy between trialists’ perception of current and optimal centre selection for RCTs: generalisability of results is rarely incorporated explicitly in trial design, despite acknowledging that this should ideally happen. Several reasons could underlie the discrepancy between current and optimal practice: first, the trial protocols included in the systematic review may be subject to incomplete reporting and thus trialists’ judgements about generalisability may have been masked. Second, there is currently little explicit guidance towards incorporating generalisability when enrolling centres. This applies both to the parameters that should be considered and to the weighting algorithm to be applied. Moreover, it is only the recent CONSORT statement extension to randomised trials of non-pharmacologic interventions [32] where generalisability with respect to centre is explicitly required when reporting trial findings; the CONSORT statements for parallel group trials [40] and cluster trials [41] are relatively vague about discussing generalisability issues. The inclusion of generalisability issues in standardised trial protocols (the SPIRIT Initiative [42]) may be a crucial step towards a sustainable improvement in the field. Third, RCTs are primarily driven by clinical outcomes, which usually assume constant treatment effect across settings, and give less importance to economic outcomes, which are most sensitive to geographical variations.

Generalisability is of direct interest to pragmatic RCTs and much less so in explanatory trials. The PRECIS tool states that in a pragmatic trial the interventions are applied “by the full range of practitioners and in the full range of clinical settings, regardless of their expertise” [15]. The implications in terms of centre selection are two-fold: from a trial design perspective, neglecting particular types of settings affects the pragmatic nature of a RCT; and from a research perspective, adequately reporting information on centre selection informs the assessment of a trial’s position on the pragmatic-explanatory continuum.

Our results show that most RCTs do not select their centres with a view to the generalisability of results, which leaves two alternatives: 1) the results *are* largely generalisable purely by chance; or 2) the results *are assumed* to be generalisable, but researchers and policy makers are unaware if this assumption holds. The problem is that the results of RCTs preferentially recruiting from a limited number of sites when hundreds of others may be available will actually inform policy recommendations for all the centres, which makes bias a legitimate concern, especially in outcomes such as cost-effectiveness. A legitimate future research direction is to develop a tool that can assess the extent of this bias.

It has to be acknowledged that the matter is primarily relevant for therapeutic areas where a choice of centres is available (e.g. GP practices, general hospitals). In the case of specialised care, where few specialist units exist, the issue becomes trivial.

### Future Research

Whilst trialists acknowledge the need for greater consideration of centre selection in order to ensure generalisability in ideal practice, a key concern is how such a change would be accomplished, particularly since the focus of centre selection is currently meeting recruitment targets. Two directions are apparent: first, there is a need for improved reporting of centre selection both in trial protocols and subsequent publications. This would enable readers to consider both the generalisability of the study population compared with the general population, but crucially also centre level characteristics (such as patient throughput) that may influence implementation and outcomes in real-life practice. Second, future research providing insights into quantitative methods of incorporating generalisability at the design stage, potentially leading towards an even more rational centre selection, can only benefit RCTs. Reviewers, journal editors and funding bodies may play a seminal role in facilitating this process.

## Supporting Information

Table S1 **Systematic review - complete results of meta-summary, including subgroup analysis by type of intervention and RCT design.**
(DOC)

Appendix S1 **Systematic review protocol.**
(DOC)

Appendix S2 **Full-length survey.**
(PDF)
