# Supplementary material for: Centre Selection for Clinical Trials and the Generalisability of Results: A Mixed Methods Study
Source: PLoS One. 2013 Feb 22;8(2):e56560. doi: 10.1371/journal.pone.0056560 (PMC3579829; doi:10.1371/journal.pone.0056560)
Supplement: Table S1 — Systematic review - complete results of meta-summary, including subgroup analysis by type of intervention and RCT design. (DOC) [file pone.0056560.s001.doc]

Table S1. Systematic review - complete results of meta-summary, including subgroup analysis by type of intervention and RCT design

|  | Number (%) of included RCTs reporting each consideration | | | | | | | | | | | | | |
| --- | --- | --- | --- | --- | --- | --- | --- | --- | --- | --- | --- | --- | --- | --- |
|  | TOTAL | | NON-PHARMACOLOGIC | | | PHARMACOLOGIC | | | CLUSTER | | | NON-CLUSTER | | |
|  | **N** | % total | **N** | % total | % group | **N** | % total | % group | **N** | % total | % group | **N** | % total | % group |
| **CENTRE SELECTION CONSIDERATIONS** | **78** | **60%** | **56** | **43%** | **58%** | **22** | **17%** | **67%** | **13** | **10%** | **93%** | **65** | **50%** | **57%** |
| **DIVERSITY AND REPRESENTATIVENESS** | **31** | **24%** | **26** | **20%** | **27%** | **5** | **4%** | **15%** | **6** | **5%** | **43%** | **25** | **19%** | **22%** |
| POPULATION CHARACTERISTICS | **14** | **11%** | **13** | **10%** | **14%** | **1** | **1%** | **3%** | **2** | **2%** | **14%** | **12** | **9%** | **10%** |
| cultural background | 1 | 1% | 1 | 1% | 1% | 0 | 0% | 0% | 0 | 0% | 0% | 1 | 1% | 1% |
| ethnicity | 9 | 7% | 8 | 6% | 8% | 1 | 1% | 3% | 1 | 1% | 7% | 8 | 6% | 7% |
| socio-economic status | 10 | 8% | 9 | 7% | 9% | 1 | 1% | 3% | 2 | 2% | 14% | 8 | 6% | 7% |
| HEALTH SERVICE DELIVERY | **15** | **12%** | **13** | **10%** | **14%** | **2** | **2%** | **6%** | **6** | **5%** | **43%** | **9** | **7%** | **8%** |
| patient case-mix | 2 | 2% | 2 | 2% | 2% | **1** | 1% | 3% | 2 | 2% | 14% | 2 | 2% | 2% |
| intervention throughput | 1 | 1% | 1 | 1% | 1% | **0** | 0% | 0% | 1 | 1% | 7% | 1 | 1% | 1% |
| organisations or practitioners | 9 | 7% | 9 | 7% | 9% | **1** | 1% | 3% | 9 | 7% | 64% | 9 | 7% | 8% |
| services offered | 4 | 3% | 4 | 3% | 4% | **0** | 0% | 0% | 4 | 3% | 29% | 4 | 3% | 3% |
| CENTRE SETTING | **15** | **12%** | **12** | **9%** | **13%** | **3** | **2%** | **9%** | **2** | **2%** | **14%** | **13** | **10%** | **11%** |
| environment | 1 | 1% | 1 | 1% | 1% | 0 | 0% | 0% | 0 | 0% | 0% | 1 | 1% | 1% |
| regions | 3 | 2% | 1 | 1% | 1% | 2 | 2% | 6% | 0 | 0% | 0% | 3 | 2% | 3% |
| size | 1 | 1% | 1 | 1% | 1% | 0 | 0% | 0% | 1 | 1% | 7% | 0 | 0% | 0% |
| plain setting | 2 | 2% | 2 | 2% | 2% | 0 | 0% | 0% | 2 | 2% | 14% | 2 | 2% | 2% |
| type of communities | 1 | 1% | 1 | 1% | 1% | 0 | 0% | 0% | 1 | 1% | 7% | 1 | 1% | 1% |
| urban vs. rural | 8 | 6% | 8 | 6% | 8% | 1 | 1% | 3% | 8 | 6% | 57% | 8 | 6% | 7% |
| **CENTRE CHARACTERISTICS** | **57** | **44%** | **39** | **30%** | **41%** | **18** | **14%** | **55%** | **7** | **5%** | **50%** | **50** | **39%** | **43%** |
| CENTRE SETTING | **4** | **3%** | **3** | **2%** | **3%** | **1** | **1%** | **3%** | **2** | **2%** | **14%** | **2** | **2%** | **2%** |
| geographical location | 2 | 2% | 1 | 1% | 1% | 1 | 1% | 3% | 1 | 1% | 7% | 1 | 1% | 1% |
| uniqueness in the region | 1 | 1% | 1 | 1% | 1% | 0 | 0% | 0% | 0 | 0% | 0% | 1 | 1% | 1% |
| deprivation status | 1 | 1% | 1 | 1% | 1% | 0 | 0% | 0% | 1 | 1% | 7% | 0 | 0% | 0% |
| HEALTH SERVICE DELIVERY ('RESEARCH-READY') | **16** | **12%** | **11** | **9%** | **11%** | **5** | **4%** | **15%** | **0** | **0%** | **0%** | **16** | **12%** | **14%** |
| centre of excellence | 1 | 1% | 1 | 1% | 1% | 0 | 0% | 0% | 0 | 0% | 0% | 1 | 1% | 1% |
| clinical interest | 6 | 5% | 2 | 2% | 2% | 4 | 3% | 12% | 0 | 0% | 0% | 6 | 5% | 5% |
| computer systems | 1 | 1% | 0 | 0% | 0% | 1 | 1% | 3% | 0 | 0% | 0% | 1 | 1% | 1% |
| Department of Health approved centre | 1 | 1% | 1 | 1% | 1% | 0 | 0% | 0% | 0 | 0% | 0% | 1 | 1% | 1% |
| links with other facilities | 3 | 2% | 2 | 2% | 2% | 1 | 1% | 3% | 0 | 0% | 0% | 3 | 2% | 3% |
| NHS centre | 7 | 5% | 6 | 5% | 6% | 1 | 1% | 3% | 0 | 0% | 0% | 7 | 5% | 6% |
| satisfactory peer review | 1 | 1% | 1 | 1% | 1% | 0 | 0% | 0% | 0 | 0% | 0% | 1 | 1% | 1% |
| INTERVENTION | **31** | **24%** | **24** | **19%** | **25%** | **7** | **5%** | **21%** | **3** | **2%** | **21%** | **28** | **22%** | **24%** |
| appropriate training | 3 | **2%** | 3 | 2% | 3% | 0 | 0% | 0% | 0 | 0% | 0% | 3 | 2% | 3% |
| suitable to implement the intervention | 16 | 12% | 12 | 9% | 13% | 4 | 3% | 12% | 0 | 0% | 0% | 16 | 12% | 14% |
| experience in delivering the intervention | 13 | 10% | 10 | 8% | 10% | 3 | 2% | 9% | 0 | 0% | 0% | 13 | 10% | 11% |
| not running the intervention | 6 | 5% | 6 | 5% | 6% | 0 | 0% | 0% | 3 | 2% | 21% | 3 | 2% | 3% |
| performance in delivering the intervention | 5 | 4% | 4 | 3% | 4% | 1 | 1% | 3% | 0 | 0% | 0% | 5 | 4% | 4% |
| RESEARCH | **19** | **15%** | **11** | **9%** | **11%** | **8** | **6%** | **24%** | **2** | **2%** | **14%** | **17** | **13%** | **15%** |
| able to support research | 2 | 2% | 2 | 2% | 2% | 0 | 0% | 0% | 0 | 0% | 0% | 2 | 2% | 2% |
| part of a research network | 10 | 8% | 4 | 3% | 4% | 6 | 5% | 18% | 1 | 1% | 7% | 9 | 7% | 8% |
| research experience | 10 | 8% | 6 | 5% | 6% | 4 | 3% | 12% | 0 | 0% | 0% | 10 | 8% | 9% |
| interest in research | 3 | **2%** | 2 | 2% | 2% | 1 | 1% | 3% | 1 | 1% | 7% | 2 | 2% | 2% |
| CENTRE SIZE | **22** | **17%** | **16** | **12%** | **17%** | **6** | **5%** | **18%** | **4** | **3%** | **29%** | **18** | **14%** | **16%** |
| catchment area | 7 | 5% | 4 | 3% | 4% | 3 | 2% | 9% | 1 | 1% | 7% | 6 | 5% | 5% |
| patient throughput | 11 | 9% | 8 | 6% | 8% | 3 | 2% | 9% | 1 | 1% | 7% | 10 | 8% | 9% |
| size of centre | 5 | **4%** | 4 | 3% | 4% | 1 | 1% | 3% | 2 | 2% | 14% | 3 | 2% | 3% |
| **TRIAL PARTICIPATION** | **37** | **29%** | **23** | **18%** | **24%** | **14** | **11%** | **42%** | **8** | **6%** | **57%** | **29** | **22%** | **25%** |
| RECRUITMENT | **17** | **13%** | **10** | **8%** | **10%** | **7** | **5%** | **21%** | **3** | **2%** | **21%** | **14** | **11%** | **12%** |
| ability to recruit | 10 | 8% | 4 | 3% | 4% | 6 | 5% | 18% | 0 | 0% | 0% | 10 | 8% | 9% |
| access to study population | 8 | 6% | 6 | 5% | 6% | 2 | 2% | 6% | 3 | 2% | 21% | 5 | 4% | 4% |
| TRIAL CONSTRAINTS | **5** | **4%** | **5** | **4%** | **5%** | **0** | **0%** | **0%** | **4** | **3%** | **29%** | **1** | **1%** | **1%** |
| proximity to study site | 2 | 2% | 2 | 2% | 2% | 0 | 0% | 0% | 2 | 2% | 14% | 0 | 0% | 0% |
| costs to trial | 2 | 2% | 2 | 2% | 2% | 0 | 0% | 0% | 1 | 1% | 7% | 1 | 1% | 1% |
| time frame of trial | 1 | 1% | 1 | 1% | 1% | 0 | 0% | 0% | 1 | 1% | 7% | 0 | 0% | 0% |
| ENSURING TRIAL PROCESSES AND REQ. | **24** | **19%** | **13** | **10%** | **14%** | **11** | **9%** | **33%** | **3** | **2%** | **21%** | **21** | **16%** | **18%** |
| arrange follow-up | 1 | 1% | 1 | 1% | 1% | 0 | 0% | 0% | 0 | 0% | 0% | 1 | 1% | 1% |
| compliance with trial procedures and regulatory requirements | 17 | 13% | 8 | 6% | 8% | 9 | 7% | 27% | 2 | 2% | 14% | 15 | 12% | 13% |
| ensuring communication | 6 | 5% | 1 | 1% | 1% | 5 | 4% | 15% | 0 | 0% | 0% | 6 | 5% | 5% |
| identify champions | 1 | 1% | 1 | 1% | 1% | 0 | 0% | 0% | 0 | 0% | 0% | 1 | 1% | 1% |
| required time, staff, facilities | 16 | 12% | 7 | 5% | 7% | 9 | 7% | 27% | 2 | 2% | 14% | 14 | 11% | 12% |
| SUPPORT | **7** | **5%** | **6** | **5%** | **6%** | **1** | **1%** | **3%** | **3** | **2%** | **21%** | **4** | **3%** | **3%** |
| support from centre management | 4 | 3% | 4 | 3% | 4% | 0 | 0% | 0% | 2 | 2% | 14% | 2 | 2% | 2% |
| support from funding bodies | 0 | 0% | 0 | 0% | 0% | 0 | 0% | 0% | 0 | 0% | 0% | 0 | 0% | 0% |
| support from local commissioners | 1 | 1% | 0 | 0% | 0% | 1 | 1% | 3% | 0 | 0% | 0% | 1 | 1% | 1% |
| support from local stakeholders | 1 | 1% | 1 | 1% | 1% | 0 | 0% | 0% | 0 | 0% | 0% | 1 | 1% | 1% |
| support from research network | 1 | 1% | 1 | 1% | 1% | 0 | 0% | 0% | 1 | 1% | 7% | 0 | 0% | 0% |
| WILLINGNESS | **9** | **7%** | **7** | **5%** | **7%** | **2** | **2%** | **6%** | **1** | **1%** | **7%** | **8** | **6%** | **7%** |
| willing to randomise | 4 | 3% | 3 | 2% | 3% | 1 | 1% | 3% | 0 | 0% | 0% | 4 | 3% | 3% |
| willingness to perform the intervention | 4 | 3% | 3 | 2% | 3% | 1 | 1% | 3% | 0 | 0% | 0% | 4 | 3% | 3% |
| willing to participate | 3 | 2% | 3 | 2% | 3% | 0 | 0% | 0% | 1 | 1% | 7% | 2 | 2% | 2% |
