# Supplementary material for: Centre Selection for Clinical Trials and the Generalisability of Results: A Mixed Methods Study
Source: PLoS One. 2013 Feb 22;8(2):e56560. doi: 10.1371/journal.pone.0056560 (PMC3579829; doi:10.1371/journal.pone.0056560)
Supplement: Appendix S1 — Systematic review protocol. (DOC) [file pone.0056560.s002.doc]

The rationale for centre selection in RCTs:

Systematic review and meta-summary

Protocol

**Objective**

To investigate the process of centre selection in RCTs with a parallel economic evaluation in the UK

**Specific questions**

How do RCT investigators report the rationale for selecting and including centres in the RCT?

**Inclusion criteria**

- multi-centre RCTs with a parallel economic evaluation. Any type of economic evaluation is accepted as long as both costs and outcome data are collected alongside the RCT.

- multinational RCTs are accepted if at least one centre in the RCT is in the UK;

- RCTs started after January 1^st^ 2005 and before January 2012.

**Exclusion criteria**

- RCTs without an explicit economic evaluation component described in the protocol;

- RCTs initiated before 1^st^ January 2005 or after January 2012;

- decision modelling studies based on one or more RCTs;

- other types of studies apart from RCTs: cohort studies, case-control studies, follow-up studies, diagnostic accuracy studies;

- studies where UK centres did not participate;

- studies that involve animal subjects;

- studies for which the protocol is not available;

- pilot RCTs or feasibility studies.

**Data sources**

The NIHR HTA Primary Research (trial) repository, available online at <http://www.hta.ac.uk/projectdata/PjtSearchResult.asp>

**Study selection**

Phase I: All the projects listed in the NIHR HTA Primary Research repository will be scanned against the inclusion/exclusion criteria based on the information in the project abstract and, if necessary, the study protocol.

Phase II: The protocols of all included RCTs and all accompanying publications, as listed on the NIHR website, will be downloaded and scanned against the inclusion/exclusion criteria.

Phase III: RCTs included from Phase II will undergo data extraction.

AG will perform Phase I and Phase II on all the projects listed in the NIHR HTA Primary Research repository. BF will perform Phase I and Phase II on a selection of 20% of the projects that will be randomly selected using the online random numbers generator [www.random.org](http://www.random.org/).

**Data extraction**

For all the included studies (Phase III) the protocol and all accompanying publications will be downloaded from the NIHR HTA Primary Research repository and scanned for relevant information.

The following information will be extracted: study authors; project start year; acronym of RCT; intervention; control; rationale for centre selection (free text); rationale for centre selection (yes/no); discussion on generalisability by location (free text); discussion on generalisability by location (yes/no/unclear).

Definitions:

Rationale for centre selection 'yes': the protocol explicitly mentions one or more reasons or considerations that justify or describe the choice of particular centres for the RCT to the detriment of others. The mere enumeration of recruiting centres does not fall into this category.

Rationale for centre selection 'no': the protocol does not identify any obvious consideration that justifies or describes the choice of particular centres for the RCT to the detriment of others.

Discussion on generalisability by location 'yes': the protocol explicitly mentions that generalisability by location will be addressed in subsequent analyses of the economic evaluation results and specifies the methods that will be employed.

Discussion on generalisability by location 'unclear': the protocol explicitly mentions that generalisability by location will be addressed in subsequent analyses of the economic evaluation results, but does not identify the methods that will be employed.

Discussion on generalisability by location 'no': the protocol does not mention explicitly that generalisability by location will be addressed in subsequent analyses of the economic evaluation results.

The protocols and accompanying publications will be downloaded in pdf format (Adobe Acrobat). The information on centre selection will be identified using the 'Find' command embedded in Adobe Reader with the following search terms entered separately: 'centre', 'site', 'clinic', 'hospital', 'practice' and any other relevant term specific to the study in question. The information on generalisability by location of economic evaluation results will be identified using the 'Find' command embedded in Adobe Reader with the following search terms entered separately: 'economic', 'cost'.

AG will perform data extraction for all the included studies. BF will check data extraction for the included studies in the random sample of 20% considered for Phase I and II.

**Data analysis**

Following the extraction of free text information relevant for the two review questions, categories will be developed using conventional content analysis to summarise the content regarding centre selection and generalisability of economic evaluation results.

AG will perform the qualitative data analysis using NVivo 8 software: this involves developing the codes and manually coding the extracted free text information in all the included studies. The information will be abstracted, reformulated and categorized. A frequency effect size will be calculated for each emerging category as the ratio between the number of studies containing a finding and the total number of included studies.

BF will check the code structure and the coding for all the included studies. Any disagreement will be resolved by discussion.

**Reporting**

The categories emerging from the meta-summary will be presented in a table together with their frequency (number of studies where they have been identified) and effect size (calculated as presented in **Data analysis**).
