# Supplementary material for: Centre Selection for Clinical Trials and the Generalisability of Results: A Mixed Methods Study
Source: PLoS One. 2013 Feb 22;8(2):e56560. doi: 10.1371/journal.pone.0056560 (PMC3579829; doi:10.1371/journal.pone.0056560)
Supplement: Appendix S2 — Full-length survey. (PDF) [file pone.0056560.s003.pdf]

UNIVERSITY OF BIRMINGHAM

Exit this survey

8%

Thank you for your interest in this research.

What is the purpose of this study?

The aim of the study is to gather information on how centre selection is carried out in randomised controlled trials (RCTs) with a parallel economic evaluation. We are approaching staff affiliated with Clinical Trials Units and Research Design Services in the UK. We want to know your views on which considerations are the most relevant when deciding on which centres are included in such a RCT.

Who is doing this research?

This research is carried out as part of a PhD studentship in health economics, based in Primary Care Clinical Sciences at the University of Birmingham. The researchers involved in the study are Dr. Melanie Calvert (main investigator) and Adrian Gheorghe, MSc. (studentship holder). The Science, Technology, Engineering and Mathematics Ethical Review Committee at the University of Birmingham have favourably reviewed the study.

How long will it take?

The questionnaire has 9 questions and its completion is expected to last about 10 minutes.

How will data collected from you be protected?

All the data collected from you will be kept anonymous and confidential. You will not be asked for any personal information (e.g. name, socio-demographic characteristics, contact details). We will ask you, though, about your professional position and your work experience. The results of the questionnaire and any reports derived from it will be securely stored on the computer systems in Primary Care Clinical Sciences at the University of Birmingham for the duration of 10 years. After this period they will be deleted so that they cannot be recovered.

Once you agree to take part, can you change your mind?

Yes, you can exit the questionnaire at any time and your answers up to that point will not be analysed. Due to anonymity, you will not be able to withdraw after submitting the answers because there is no way we can retrieve your individual answers.

Who can you contact should you want to ask questions?

Primary Care Clinical Sciences, University of Birmingham
Birmingham B15 2TT, UK
Dr. Melanie Calvert
m.calvert@bham.ac.uk
t: 0121 4148595

Mr. Adrian Gheorghe
axg986@bham.ac.uk

t: 0121 4143951

NOTE: Advancing to the next page is equivalent to your giving CONSENT to have your answers analysed. Anonymity and confidentiality will be ensured.

Next

Powered by **SurveyMonkey**
Create your own free online survey now!

Please consider a **phase III multi-centre RCT** with a parallel **economic evaluation** (within-trial economic evaluation).

A 'centre' can be defined broadly, depending on the intervention. Examples of centres include, but are not limited to: GP practices, clinics, hospitals, tertiary centres, neighbourhoods or entire cities.

'Parallel economic evaluation' refers to the collection of cost and outcome data (e.g. health-related quality of life information) alongside the RCT.

In the following questions you will be asked about various considerations influencing the decision to include a centre in a RCT.

Please ASSUME in all cases that any given centre fulfils two basic requirements:
1. The centre has enough qualified staff, physical space and relevant equipment available for the RCT.
2. The centre has access to the relevant study population.

Powered by **SurveyMonkey**
Create your own free online survey now!

UNIVERSITY OF BIRMINGHAM

Exit this survey

23%

The following 4 questions are about your CURRENT practice.

Prev Next

Powered by **SurveyMonkey**
Create your own free online survey now!

UNIVERSITY OF BIRMINGHAM

Exit this survey

31%

∗1. YOUR CURRENT PRACTICE: When considering the inclusion of a centre in a RCT with a parallel economic evaluation, which centre characteristics do you usually look for?

Please choose the most important characteristics from the list below. No explicit ranking is required. Please select a MINIMUM of 3 and a MAXIMUM of 5 answers.

- Local clinical staff understand the methodological underpinnings of RCTs.
- The geographical location of the centre is convenient for logistical reasons.
- The centre belongs to a relevant research network.
- Local staff have had experience with conducting RCTs in the past.
- The centre's computer systems are compatible with the trial centre's computer systems.
- The centre retains or contributes to generalisability in terms of clinical practice.
- The centre has support from local commissioners to participate in the RCT.
- The centre retains or contributes to generalisability in terms of population characteristics.
- There is a good communication relationship between the trials unit and centre staff.
- The centre is able to obtain necessary approvals (including R&D) in a timely manner.
- The centre is able to recruit the desired number of patients in a timely manner.
- The centre staff show a degree of interest in the RCT.
- The centre retains or contributes to generalisability in terms of economic evaluation results.
- Other (please complete below).

150 characters limit

Prev Next

Powered by SurveyMonkey
Create your own free online survey now!

UNIVERSITY OF BIRMINGHAM

Exit this survey

38%

*2. YOUR CURRENT PRACTICE: What do you think has the most influence on a centre's enrolment in a RCT with a parallel economic evaluation?

Please choose the most important considerations from the list below. No explicit ranking is required. Please select a MINIMUM of 3 and a MAXIMUM of 5 answers.

- The recruiting time frame of the RCT
- The state of the local research environment (e.g. competing RCTs, trial fatigue)
- The centre staff know the Chief Investigator.
- Patient convenience i.e. travel distance to the centre, additional costs (e.g. parking) etc.
- Characteristics of the RCT design: type of intervention, sample size, number of centres required etc.
- The budget of the RCT
- The extent to which local staff are motivated to participate in the RCT
- The type of geographical setting where the centre is located (rural vs. urban)
- The efficiency of the local R&D department at issuing approvals
- Requirements of funding and regulatory bodies (e.g. Cancer Research UK, NIHR)
- The rarity of the disease under investigation
- Other (please complete below)

150 characters limit

Prev Next

Powered by SurveyMonkey
Create your own free online survey now!

UNIVERSITY OF BIRMINGHAM

Exit this survey

46%

3. YOUR CURRENT PRACTICE: In your opinion, who drives the centre enrolment process in a RCT with a parallel economic evaluation?

Please choose a maximum of 2 answers from the list below.

- Trial health economist
- Trial coordinator/Trial manager
- Trial Management Group members as a team
- Chief Investigator
- Data Monitoring Committee members
- Trial statistician
- Research networks
- Other (please complete below)

150 characters limit

Prev Next

Powered by SurveyMonkey
Create your own free online survey now!

UNIVERSITY OF BIRMINGHAM

Exit this survey

54%

＊4. YOUR CURRENT PRACTICE: Would you say that health economics considerations influence the decision to include a centre in a RCT with a parallel economic evaluation?

Yes, but only to a limited extent.

Not at all.

Yes, to a great extent.

If you have chosen either of the options starting with 'Yes', please could you explain in more detail why you gave this answer? (1000 character limit)

Prev Next

Powered by **SurveyMonkey**
Create your own free online survey now!

UNIVERSITY OF BIRMINGHAM

Exit this survey

69%

∗5. OPTIMAL PRACTICE: When considering the inclusion of a centre in a RCT with a parallel economic evaluation, which centre characteristics do you think should IDEALLY be sought?

Please choose the most important characteristics from the list below. No explicit ranking is required. Please select a MINIMUM of 3 and a MAXIMUM of 5 answers.

The geographical location of the centre is convenient for logistical reasons.

The centre's computer systems are compatible with the trial centre's computer systems.

Local clinical staff understand the methodological underpinnings of RCTs.

The centre retains or contributes to generalisability in terms of economic evaluation results.

The centre staff show a degree of interest in the RCT.

The centre is able to recruit the desired number of patients in a timely manner.

The centre retains or contributes to generalisability in terms of clinical practice.

The centre is able to obtain necessary approvals (including R&D) in a timely manner.

There is a good communication relationship between the trials unit and centre staff.

The centre retains or contributes to generalisability in terms of population characteristics.

The centre has support from local commissioners to participate in the RCT.

The centre belongs to a relevant research network.

Local staff have had experience with conducting RCTs in the past.

Other (please complete below)

150 characters limit

Prev Next

Powered by SurveyMonkey
Create your own free online survey now!

UNIVERSITY OF BIRMINGHAM

Exit this survey

77%

***6. OPTIMAL PRACTICE: Which considerations do you think should IDEALLY have the most influence on the practice of enrolling a centre in a RCT with a parallel economic evaluation?**

Please choose the most important considerations from the list below. No explicit ranking is required. Please select a MINIMUM of 3 and a MAXIMUM of 5 answers.

- The type of geographical setting where the centre is located (rural vs. urban)
- The rarity of the disease under investigation
- Characteristics of the RCT design: type of intervention, sample size, number of centres required etc.
- The recruiting time frame of the RCT
- The centre staff know the Chief Investigator.
- Patient convenience i.e. travel distance to the centre, additional costs (e.g. parking) etc.
- The extent to which local staff are motivated to participate in the RCT
- The budget of the RCT
- Requirements of funding and regulatory bodies (e.g. Cancer Research UK, NIHR)
- The efficiency of the local R&D department at issuing approvals
- The state of the local research environment (e.g. competing RCTs, trial fatigue)
- Other (please complete below)

150 characters limit

Prev Next

Powered by SurveyMonkey
Create your own free online survey now!

UNIVERSITY OF BIRMINGHAM

Exit this survey

85%

7. OPTIMAL PRACTICE: In your opinion, which of the following should IDEALLY drive the centre enrolment process in a RCT with a parallel economic evaluation?

Please choose a maximum of 2 answers from the list below.

Trial Management Group members as a team

Chief Investigator

Trial coordinator/Trial manager

Data Monitoring Committee members

Research networks

Trial statistician

Trial health economist

Other (please complete below)

150 characters limit

Prev Next

Powered by SurveyMonkey
Create your own free online survey now!

UNIVERSITY OF BIRMINGHAM

Exit this survey

92%

Please give us a bit of information about yourself.

***8. What is your professional role within the trials unit? Please state your PRIMARY role if you have more than one professional position.**

Clinical investigator

Statistician

Trial coordinator/Trial manager

Health economist

Clinical trials methodologist

Epidemiologist

Evidence synthesis expert

Qualitative researcher

Outcomes research expert

Other academic position (e.g. research associate, research fellow, senior research fellow)

Other (please complete below)

150 characters limit

***9. How long have you been involved in the design and/or conduct of RCTs?**

Less than 2 years

Between 2 and 5 years

Between 5 and 10 years

More than 10 years

Prev Next

Powered by SurveyMonkey
Create your own free online survey now!

UNIVERSITY OF BIRMINGHAM

Exit this survey

100%

THANK YOU for taking the time to complete this questionnaire.

10. If you have any feedback or comments about this questionnaire and/or the nature of this research, please write them below. Your input is highly valued.

Prev SUBMIT

Powered by **SurveyMonkey**
Create your own free online survey now!
